# Nephrotoxicity and highly active antiretroviral therapy: Mitigating action of *Momordica charantia*

**DOI:** 10.1016/j.toxrep.2018.09.003

**Published:** 2018-09-20

**Authors:** Ugochukwu Offor, Edwin Coleridge Naidu, Oluwatosin Olalekan Ogedengbe, Ayoola Isaac Jegede, Aniekan Imo Peter, Onyemaechi Okpara Azu

**Affiliations:** a Department of Clinical Anatomy, School of Laboratory Medicine and Medical Sciences, Nelson R Mandela School of Medicine, University of KwaZulu-Natal, South Africa; b Department of Anatomy, College of Medicine and Health Sciences, Afe Babalola University, Ado Ekiti, Nigeria; c Department of Anatomy, Faculty of Basic Medical Sciences, University of Uyo-Nigeria, Nigeria; d Department of Anatomy, School of Medicine, University of Namibia, Windhoek, Namibia

**Keywords:** DTNB, 5, 5'-dithiobis-(2-nitrobenzoic acid), 6-HD, 6-hydroxydopamine, AIDS, acquired immune deficiency syndrome, ALB, albumin, ANOVA, analysis of variance, AREC, animal research ethics committee, BRU, Biomedical Resource Unit, BGL, blood glucose levels, BUN, blood urea nitrogen, BW, body weight, CAT, catalase, DNA, deoxyribonucleic acid, DETAPAC, diethylenetriamine – penta acetic acid, GSH, reduced glutathione, H and E, haematoxylin and eosin, HAART, highly active antiretroviral therapy, HIV, human immunodeficiency virus, KW, kidney weight, KWBR, kidney weight body ratio, LPO, lipid peroxidation, MDA, malondialdehyde, MT, Masson’s Trichome, *M. charantia*, *Momordica charantia*, NRTIs, nucleoside reverse transcriptase inhibitors, PLWHA, people living with HIV and AIDS, PAS, Periodic Acid Schiff, PBS, phosphate buffer solution, ROS, reactive oxygen species, rpm, revolutions per minute, SCr, serum creatinine, SDS, sodium dodecyl sulfate, SOD, superoxide dismutase, SD, standard deviation, TBARS, thiobarbituric acid reactive substances, TCA, trichloroacetic acid, UKZN, University of KwaZulu Natal, HAART, Nephrotoxicity, Kidney, *Momordica charantia*, Histopathology, Sprague-Dawley rats

## Abstract

*Momordica charantia* (*M. charantia*) is known for its antioxidant and antidiabetic properties. The aim of this study is to investigate the renoprotective effects of *M. charantia* in rats following treatment with highly active antiretroviral therapy (HAART) regimen triplavar. Adult male Sprague-Dawley rats weighing 178.1–220.5 g (n = 36) were divided into six groups (A–F) with each group comprising of six (n = 6) rats. The drugs and extract were administered via oral gavage. The therapeutic dose of triplavar was adjusted using the human therapeutic dose equivalent for the rat model. Animals were euthanized on the tenth week with kidneys removed for examination and blood obtained via cardiac puncture. Levels of oxidative stress enzymes (superoxide dismutase-SOD, catalase-CAT, and reduced glutathione-GSH) were significantly lowered in all groups not receiving *M. charantia*. The levels of thiobarbituric acid reactive substances (TBARS) were increased resulting in free radical formation via auto-oxidation. Renal parameters showed no albuminuria, normal blood urea nitrogen (BUN), serum creatinine (SCr) and electrolytes in groups treated with *M. charantia*. HAART treated (Group B) showed severe albuminuria, a significantly (*p < 0.05*) raised BUN and SCr and gross electrolyte disturbances. Blood glucose levels were significantly raised in groups not receiving the adjuvant *M. charantia* (*p < 0.05*). Histopathology in HAART treated animals showed glomerular capillary abnormalities and cellular infiltrations while *M. charantia* treated animals showed an essentially normal glomerular appearance with capillary loops and normal cytoarchitecture. In conclusion *M. charantia* extract administration improved blood glucose levels, restored renal histology, reinstate renal function, reduce body weight loss and restores hyperglycemia.

## Highlights

• Highly active antiretroviral therapy (HAART) associated nephrotoxicity is characterized by tubular necrosis with glomerular hypertrophy.
• Derangements of the cytoarchitectural patterns of the kidney were seen as a result of treatment with HAART regimen (triplavar).
• Co-administration of *M. charantia* and HAART mitigates the intensive histopathological changes of the rat kidney.
• Nephrotoxicity is associated with increased oxidative stress which stems from increased reactive oxygen species (ROS) generation in tissues.
• *M. charantia* possesses phytochemical properties which are powerful counter measures against oxidative stress tissue damage.

## Graphical abstract

**Figure:**
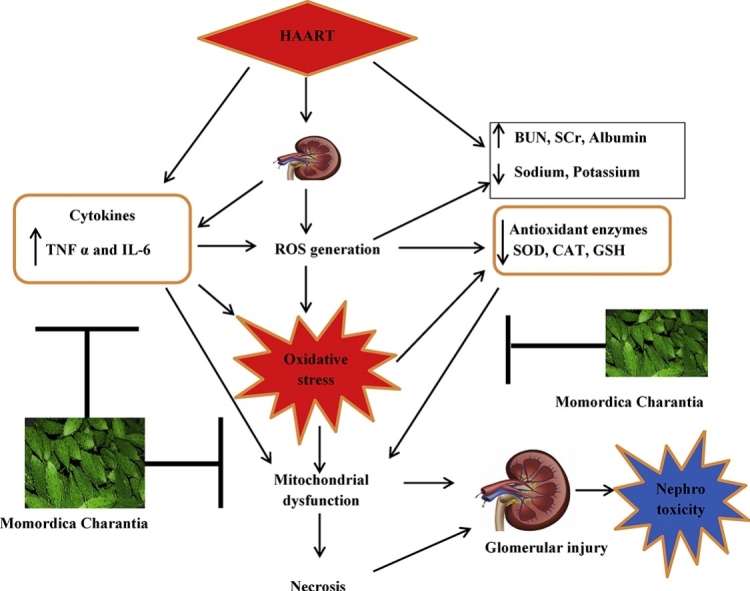

## 1 Introduction

The successful introduction of highly active antiretroviral therapy (HAART) for the management of human immunodeficiency virus (HIV) and acquired immune deficiency syndrome (AIDS) significantly increased the life expectancy among HIV-infected patients with unprecedented changes in disease progression and mortality [1]. However, despite this full control, empirical evidence still demonstrates that nephrotoxicity is increasing rapidly among people living with HIV and AIDS (PLWHA) thus dampening the perceived impact of HAART. Nephrotoxicity results from mitochondrial dysfunction induced by nucleoside reverse transcriptase inhibitors (NRTIs) since they inhibit reverse transcriptase of HIV along with inhibition of mitochondrial deoxyribonucleic acid (DNA) polymerase from host cells with subsequent deficits in mitochondrial DNA encoded enzymes [2]. It is characterized by tubular necrosis with glomerular hypertrophy, which is instigated by mesangial cell proliferation and excessive accumulation of the extracellular matrix [3].

Nephrotoxicity is strongly associated with increased oxidative stress which stems from increased reactive oxygen species (ROS) generation in tissues and a depletion of natural enzyme antioxidants expression and activity [4]. ROS through the formation of DNA adducts damages the DNA causing loss of DNA repair mechanism and induce gene activation of oxidative-stress responsive maladaptive pathways thereby promoting apoptosis in glomerular cells which leads to characteristic morphological and functional abnormalities associated with nephropathy [4]. Strategies that reduce ROS and improve functional capacity of the kidney will provide therapeutic benefits in PLWHA under HAART.

One plant that has received so much attention for its array of phytochemical properties is *Momordica charantia (M. charantia). M. charantia* is an economically important medicinal plant belonging to the family *Cucurbitaceae* known as balsam pear or karela. It is a tropical vegetable which is a common food in Indian cuisine and has been used extensively in folk medicine as a remedy for a number of diseases and disorders. Isolated phytochemicals of *M. charantia* has been documented with in vitro antioxidant and antiviral activity against numerous viruses including *Epstein-Barr*, herpes and HIV viruses [5] In an in vivo study, *M. charantia* leaf extract demonstrated the ability to increase resistance to viral infections as well as to provide an immunostimulant effect in humans and animals (increasing interferon production and natural killer cell activity). Two proteins known as alpha-and beta- momorcharin (which are present in the seeds, fruit, and leaves) have been reported to inhibit the HIV virus in vitro [5].

But its efficacy in mitigating adverse biochemical defects and structural configurations of the kidney that arise as a result of antiretroviral therapy is unknown. Hence, the present investigation was postulated to explore the mechanism of renoprotective nature of *M. charantia* following antiretroviral therapy in adult male Sprague-Dawley rats.

## 2 Materials and methods

### 2.1 Materials

HAART regimen- Triplavar *(Cipla-Medpro)* containing Lamivudine 150 mg, Nevirapine 400 mg and Zidovudine 300 mg, was used for this study. The drug was obtained from Pharmed pharmaceuticals, Pty (Ltd) Durban, South Africa. Fifty kilograms of the fresh mature unripe fruit of *M. charantia* was purchased from the local Durban markets. Samples were authenticated at the herbarium unit of the Department of Life Sciences, University of KwaZulu-Natal, Durban, South Africa (voucher no. 4617). A total of thirty-six (36) adult male Sprague-Dawley rats weighing 178.1–220.5 grams were used for the study. Ethical approval was obtained from University of KwaZulu Natal (UKZN) animal research ethics committee (AREC) – ethics number AREC/033/016D. The study was conducted at the Biomedical Resource Unit (BRU) of UKZN.

### 2.2 Methods

#### 2.2.1 Preparation of *M. charantia* fruit ethanolic extract

The fruits were cleaned, sliced into small pieces and the seeds separated out and discarded. The sliced green fruit was first weighed and then dried in shade for approximately 2 weeks. It was then weighed again to obtain the final dry weight before pulverizing into a fine powder in a commercial grinder and stored at 5 °C until ready for extraction. The active ingredients were obtained by Soxhlet extraction using ethanol as the solvent. The solvent was evaporated in a rotary evaporator at 40°−50 °C with a percentage yield of 85.25%. The wet residue was filtered through a Whatman filter paper and the concentrated extract was stored at 4 °C until ready for use.

### 2.3 Experimental design

All rats were housed in well ventilated plastic cages [3 rats per cage (in 12 cages)] having dimensions of (52 cm long × 36 cm wide and 24 cm high) and soft wood shavings employed as beddings in the cages. They were maintained under standardized animal house conditions (temperature: 23–25 °C; light: approximately 12 h natural light per day) and were fed with standard rat pellets from (Meadow feeds a Division of Astral Operations Limited, Durban, South Africa) and given tap water ad libitum. The initial body weight of the animals was recorded before treatment and randomly distributed to the treatment groups with 6 animals per group.

Group A received Normal saline (Control)

Group B received Triplavar

Group C received *M. charantia* (200 mg/kg bw)

Group D received *M. charantia* (400 mg/kg bw)

Group E received Triplavar + *M. charantia* (200 mg/kg bw)

Group F received Triplavar + *M. charantia* (400 mg/kg bw)

The therapeutic dose of triplavar was adjusted using the human therapeutic dose equivalent for the rat model. Doses were administered via oral gavage daily for 6 days with 1day rest over a 10 week period. Animals were euthanized on day 70 by bilateral pneumothorax under anesthesia with an overdose of halothane. Blood was collected by intra-cardiac puncture and tissues were harvested for preparation of light microscopy.

### 2.4 Measurements of body weight and collection of urine samples

All experimental animals were weighed weekly by a digital scale (Mettler-Toledo, 200). For collection of urine, the rats were placed in metabolic cages for 24 h and provided with rat chow and water. The urine volume was measured and urine centrifuged to separate out debris. Urine samples were kept at −80 °C until further analysis. This procedure was done at weeks 3, 6 and 9 during the 10-week experimental period.

### 2.5 Measurement of blood glucose concentration

Blood samples were obtained from the tail using sterile needle prick. Glucose levels were measured once a week during the 10 weeks treatment using the one-touch ultra-glucometer (Boehringer-Mannheim, Germany).

### 2.6 Measurement of oxidative stress parameters and lipid peroxidation

Blood was collected in plain tubes via cardiac puncture and allowed to clot. It was then centrifuged at 3000 rpm (rpm) for 15 min and the serum decanted into Eppendorf tubes and stored at −20 °C for subsequent use. Serum was assayed for lipid peroxidation (LPO), reduced glutathione (GSH) level, superoxide dismutase (SOD) and catalase activities (CAT).

### 2.7 Serum lipid peroxidation levels

This was measured using a complex formed from the reaction between malondialdehyde (MDA) and thiobarbituric acid (TBARS) as described by [6]. Into an assay mixture containing 200 μL of 8.1% sodium dodecyl sulfate (SDS), 750 μL of 20% acetic acid (pH, 3.5), 2 mL of 0.25% TBARS and 850 μL of distilled water. 200 μL of sample of MDA standard series (0, 7.5, 15, 22.5, and 30 μM) was added in a pyrex screw-capped test tube. The mixture was heated at 95 ^O^C for 60 min in a sand bath, cooled down to room temperature and absorbance was read at 532 nm in a spectrophotometer (UVmini-1240, Shimadzu Japan). Thiobarbituric acid reactive substances (TBARS) concentrations of samples were extrapolated from MDA standard curve.

### 2.8 Serum reduced glutathione concentration

Reduced glutathione (GSH) concentration was measured in serum according to methods modified from [7]. The sample was first precipitated with 10% Trichloroacetic acid (TCA) and then centrifuged at 2000 rpm for 10 min at 25 °C. The reaction mixture contained 400 μL of supernatant, 200 μL of 0.5 M 5,5′-dithiobis-(2-nitrobenzoic acid) (DTNB) and 1.2 mL of 0.2 M sodium phosphate buffer (pH, 7.8). Absorbance was measured at 415 nm after 15 min incubation at 25 °C and GSH concentrations of samples were extrapolated from a standard curve of GSH.

### 2.9 Anti-oxidant enzyme activities

#### 2.9.1 Superoxide dismutase

Superoxide dismutase (SOD) activity was assayed according to the method of [8]. A 15 μL of 1.6 mm 6-hydroxydopamine (6-HD) was added to an assay mixture containing 170 μL of 0.1 mm diethylenetriamine – penta acetic acid (DETAPAC) in 50 mm sodium phosphate buffer (pH, 7.4) and 15 μL of sample (serum) containing 0.1 μg/μL of protein was used to start the reaction. The linear increase in absorbance was monitored at 490 nm for 5 min at 25 °C. One unit of enzyme activity was defined as the amount of enzyme required to oxidize 1 μmoL of 6-HD/min/μg protein.

#### 2.9.2 Catalase

Catalase (CAT) activity was measured using the method described by [8]. Into an assay mixture containing 340 μL of assay buffer (50 mm potassium phosphate buffer, pH 7.0) and 150 μL of 10 mm H_2_O_2_, 10 μL of a sample containing 0.1 μg/μL protein was added to start the reaction. The linear increase in absorbance was monitored at 240 nm for 5 min at 25 °C. One unit of enzyme activity was expressed as the amount of enzyme needed to decompose 1 mmoL of H_2_O_2_ /min/μg protein.

#### 2.9.3 Assessment of renal function

Serum was used for the estimation of blood urea nitrogen (BUN) and serum creatinine (SCr) using Beckman Coulter Synchron® system(s) BUN and SCr assay kit. Beckman Coulter Synchron® system BUN assay kit and Beckman Coulter Synchron® system SCr assay kit were obtained from Global Viral Laboratory, Durban, South Africa

#### 2.9.4 Tissue preparation for light microscopy

Kidneys were weighed and examined for gross pathology. A phosphate buffer solution (PBS) was used to wash out blood before preparation for tissue fixation. They were sectioned at 4 μm thickness using Leica RM 2255 microtome. Tissue was stained with Haematoxylin and Eosin (H and E), Periodic Acid Schiff (PAS) and Masson’s Trichome (MT). Slides were digitally scanned using a Leica SCN 400 (Leica Microsystems GmbH, Wetzlar, Germany) and measurements were done at 200 magnification using image analyzer Leica (DMLB) and Leica QWIN software.

### 2.10 Statistical analyses

Analyses were carried out using one-way analysis of variance, (ANOVA) followed by Dunnet’s multiple comparison *post-hoc* tests using Graph pad prism ® statistical software version 5.02. Values were expressed as mean ± standard deviation (SD) and all results tested for significance at the 95% confidence level (*p < 0.05*).

## 3 Results

### 3.1 Body weight and Organ (kidney) weight

While there was an overall increase in body weight (BW) in all groups, Groups, C and E recorded maximal significant increase in trend at *p < 0.05* for BW gained with the percentage BW gain recording 62.98% and 63.55% respectively, but however, lower when compared to control. Group B recorded the least BW gain significant at *p < 0.05* with percentage BW gain recording 44.94%. Mean kidney weight corresponds with the percentage BW gained across groups with the control group having an optimal mean kidney weight (Table 1).

**Table 1:** Body weight and kidney weight of animals.

Groups	Initial BW	Final BW	Mean BW	BW difference	BW Diff in %	Mean KW	KBWR
A	178.1	351.5	264.8 ± 122.6	173.4	65.48	2.59 ± 0.19	0.98
B	220.5	348.3	284.4 ± 90.39	127.8	44.94*	1.96 ± 0.18	0.69
C	184.3	353.8	269.1 ± 119.8	169.5	62.98	2.04 ± 0.28	0.76
D	199.2	361.2	280.2 ± 144.6	169	60.31	2.16 ± 0.17	0.97
E	180.2	348.1	264.2 ± 118.7	167.9	63.55*	2.11 ± 0.17	0.08
F	186.8	347.7	267.3 ± 113.8	160.9	60.19	2.04 ± 0.15	0.76

Values are expressed as mean ± SD for each group and considered statistically significant at p < 0.05*. BW is body weight of rats; KW is kidney weight of rats; KWBR is kidney weight body ratio. KWBR = *(Mean KW/ Mean BW) x 100.* BW diff in % = *(BW diff/ Mean BW) x 100.*

### 3.2 Blood glucose levels

**Fig. 1:**
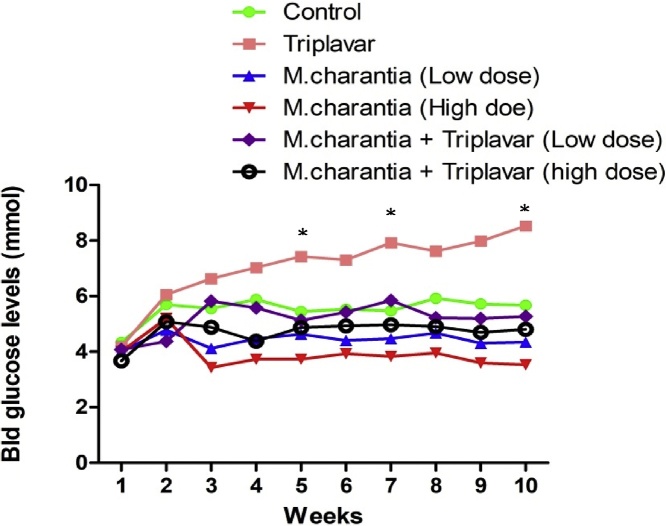
Graphical representation of blood glucose levels.

Baseline fasting blood glucose showed normal glycemia. However, from the 5th to 10th week, blood glucose levels (BGL) were significantly higher (*p < 0.05*) in triplavar treated group. Treatment with *M. charantia* alone and its concomitant use with triplavar maintained normal BGL and this was sustained between 4th and 6th week. (Fig. 1).

### 3.3 Urine parameters

Urine was collected at weeks (3, 6 and 9) for the 10 weeks duration of study for the measurement of urinary parameters (micro albuminuria, creatinine, sodium, potassium and urea). From the result, there were no significant changes in all groups (Table 2). There were also no statistical changes observed in week 6 in all groups. However, from the 9th week, the urine parameters test for animals in group B showed significant changes (*p < 0.05*). It was observed at this stage a marked increase in albumin and poor creatinine clearance, the albumin-creatinine ratio was significantly higher at this stage, (Table 3). Sodium, potassium, and urea were also significantly (*p < 0.05*) reduced. Adjuvants treatment with *M. charantia* fruit extract in all weeks of monitoring seemingly showed normal urinalysis test results when compared with control (groups C, D, E, and F).

**Table 2:** Urine Test (Week 3).

Groups	Alb (mg/L)	Creat (mmol/L)	Alb/crt ratio (mg/mmol)	Sodium (mmol/L)	Potassium (mmol/L)	Urea (mmol/L)
A	1.73 ± 0.28	9.60 ± 1.21	0.18 ± 0.05	279.30 ± 19.01	251.3 ± 13.43	411.4 ± 91.45
B	1.9.50 ± 5.18	7.56 ± 1.00	0.65 ± 1.80	261.33 ± 16.51	259.97 ± 22.25	340.4 ± 27.95
C	1.87 ± 0.23	8.96 ± 1.32	0.23 ± 0.06	268.0 ± 16.37	247.3 ± 8.95	358.9 ± 90.78
D	4.40 ± 0.46	8.53 ± 0.93	0.52 ± 0.08	265.7 ± 26.08	262.1 ± 28.86	361.2 ± 52.56
E	3.76 ± 1.10	8.23 ± 1.36	0.48 ± 0.22	268.3 ± 14.50	257.2 ± 14.70	409.8 ± 89.55
F	3.73 ± 0.77	8.83 ± 1.00	0.42 ± 0.05	258.0 ± 17.35	257.7 ± 11.71	471.8 ± 53.54

Values are expressed as means ± SD of each group. Alb is Albumin; Creat is Creatinine; Alb/crt ratio is Albumin creatinine ratio.

**Table 3:** Urine Test (Week 9).

Groups	Alb (mg/L)	Creat (mmol/L)	Alb/crt ratio (mg/mmol)	Sodium (mmol/L)	Potassium (mmol/L)	Urea (mmol/L)
A	1.80 ± 0.10	9.20 ± 1.87	0.19 ± 0.03	274.0 ± 1.00	290.0 ± 29.72	522.0 ± 44.54
B	11.63 ± 2.27*	1.53 ± 0.32*	7.70 ± 1.78	78.0 ± 10.15	78.33 ± 13.80	158.3 ± 32.87
C	2.13 ± 0.15	8.60 ± 2.76	0.26 ± 0.07	270.7 ± 35.02	264.0 ± 21.66	373.7 ± 216.6
D	1.53 ± 0.40	7.87 ± 2.73	0.21 ± 0.08	239.7 ± 30.99	256.0 ± 23.43	365.0 ± 114.0
E	1.43 ± 0.55	9.50 ± 2.36	0.15 ± 0.02	272.7 ± 24.11	311.0 ± 23.26	385.7 ± 82.31
F	1.20 ± 0.10	9.76 ± 1.68	0.12 ± 0.02	286.7 ± 9.71	308.0 ± 10.82	409.7 ± 33.65

Values are expressed as means ± SD of each group. Alb is Albumin; Creat is Creatinine; Alb/crt ratio is Albumin creatinine ratio.

### 3.4 Blood urea nitrogen (BUN) and serum creatinine (SCr) levels

Group B exhibited a significantly higher SCr and BUN (*p < 0.05*) compared to control and other experimental groups (Table 4). Adjuvant uses of *M. charantia* at both low and high doses were found to be effective in lowering SCr and BUN in all groups receiving the extract. However, they did not show any significant difference when compared with controls.

**Table 4:** BUN and SCr levels.

Groups	BUN (mmol/L)	SCr (mmol/L)
A	1.00 ± 0.26	0.96 ± 0.25
B	9.20 ± 1.60	16.67 ± 3.51*
C	1.93 ± 0.32	2.10 ± 1.11
D	1.30 ± 0.10	2.23 ± 0.25
E	1.33 ± 0.73	1.86 ± 0.15
F	1.73 ± 0.85	2.20 ± 0.10

Values are expressed as means ± SD of each group. * *p < 0.05* BUN is blood urea nitrogen; SCr is serum creatinine.

### 3.5 Lipid peroxidation and oxidative stress

**Fig. 2:**
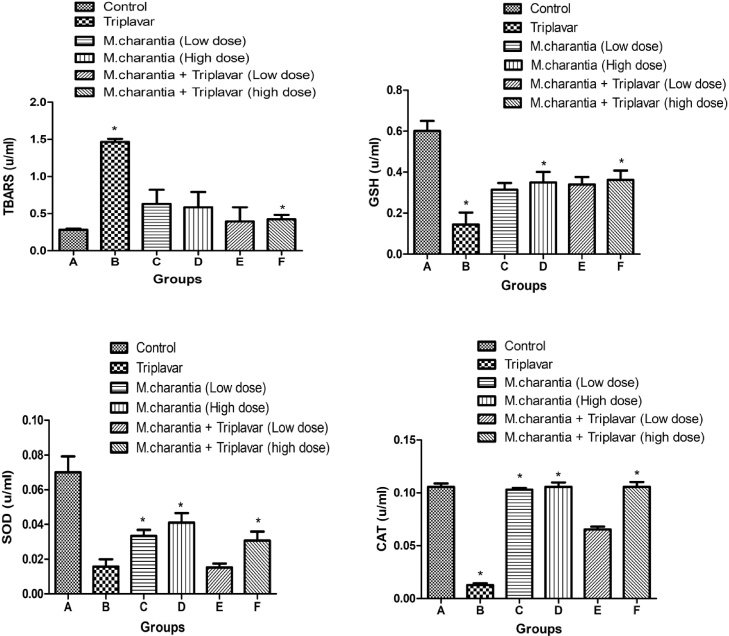
Oxidative stress measurements: Bars indicate mean ± SD, *p < 0.05.

Levels of TBARS in group B were significantly (*p < 0.05*) elevated indicating disturbances in the normal redox state. Adjuvant treatments with *M. charantia* restored these disturbances to near normal. Activities of GSH, SOD and CAT were in all decreased in Group B (HAART-regimen). These activities were significantly raised (*p < 0.05*) in groups administered with *M. charantia* alone and its concomitant administration with HAART-regimen (triplavar). Concomitant administration of *M. charantia* and triplavar at a low dose in group E did not show any significant improvement in SOD activity (Fig. 2).

### 3.6 Histopathological findings

#### 3.6.1 Haematoxylin and eosin (H and E) stains

**Fig. 3:**
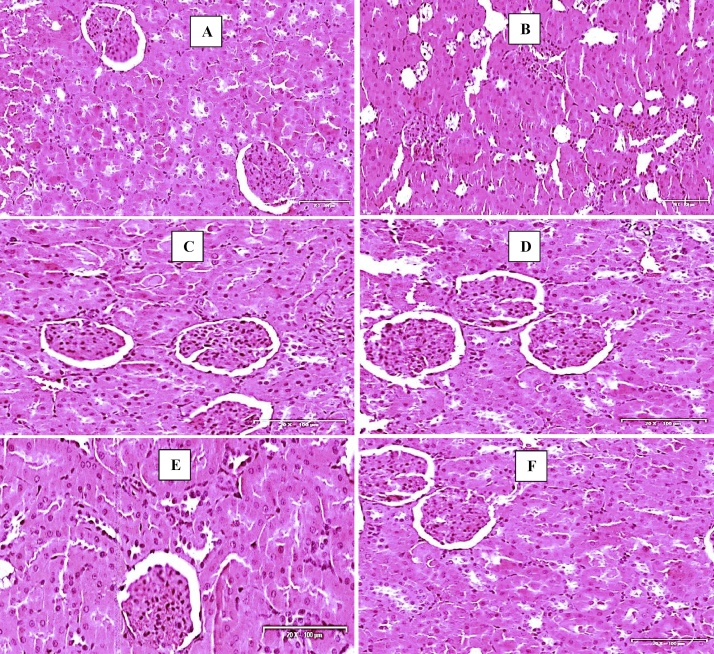
Photomicrographs of the kidney (H and E stains). Scale bar x 200 μm. (A) Control- normal structure of the kidney. (B) Triplavar treated- degeneration of the glomerulus and vacuolation of tubules (C) M. *charantia* extract Low dose- restoration of interstitium (D) M. *charantia* extract high dose- histoarchitecture essentially normal (E) M. *charantia* extract low dose and Triplavar- histoarchitecture essentially normal (F) M. *charantia* extract high dose and Triplavar- histoarchitecture essentially normal.

Histology of the kidney of the control group was essentially normal without any cellular distortions. Tubular epithelium was distinct and adequately maintained with the distinct regular lumen. The kidneys of triplavar treated animals show obliteration of glomerular capillary loops and occlusion of the capillary walls with many indistinguishable tubules, inflammation of the interstitial, epithelial desquamation and extensive necrosis of the vascular wall. Groups treated with *M. charantia* extract and its co-administration with triplavar showed reversal of the degenerative changes observed with the triplavar alone treated groups. The glomeruli were intact with no inflammatory changes in the cytoarchitectural patterns (see Fig. 3). These were the characteristics findings of the H and E stains.

#### 3.6.2 Periodic Acid Schiff (PAS) stains

**Fig. 4:**
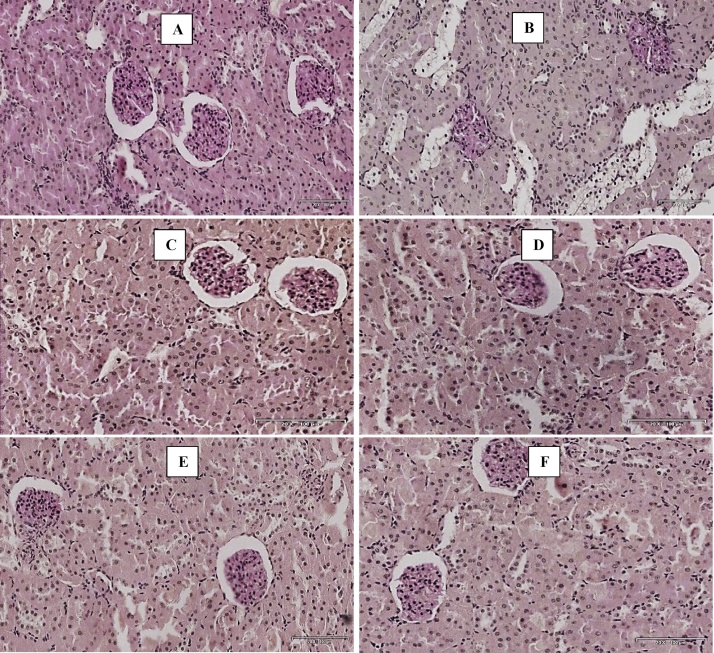
Photomicrographs of the kidney (Periodic Acid Schiff stains). Scale bar × 200 μm. (A) Control- normal structure of the kidney. (B) Triplavar treated- mucus substances and high proportion of carbohydrate macromolecules (glycogen, glycoproteins) (C) M. *charantia* fruit extract Low dose- histoarchitecture normal (D) M. *charantia* fruit extract high dose- histoarchitecture essentially normal (E) M. *charantia* extract low dose and Triplavar- histoarchitecture essentially normal (F) M. *charantia* fruit extract high dose and Triplavar- histoarchitecture essentially normal.

Triplavar treated Groups showed vacuolation of tubules characterized by high proportion of carbohydrates such as glycogen and glycoproteins and mild deposition of polysaccharides while *M. charantia* treated groups showed an essentially normal glomerular appearance with normal cytoarchitecture comparable to control (see Fig. 4).

#### 3.6.3 Masson’s Trichome (MT) stains

**Fig. 5:**
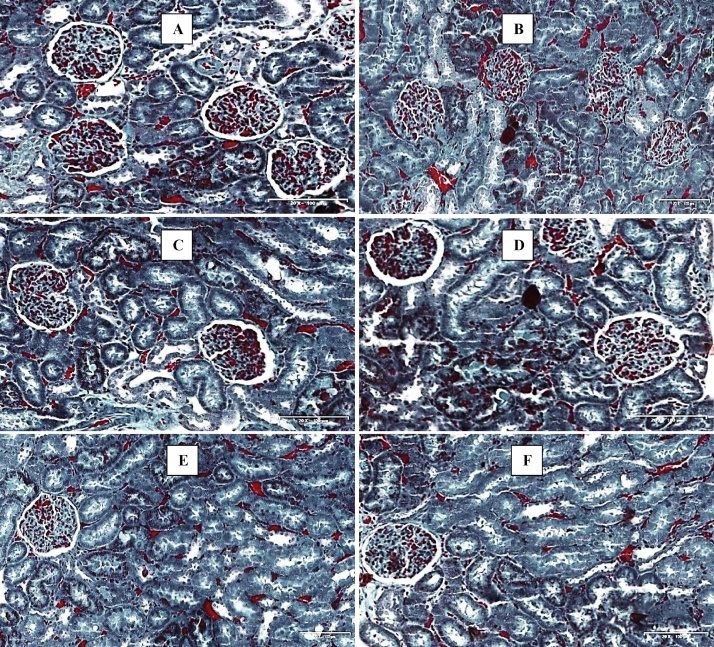
Photomicrographs of the kidney (Massons Trichome stains). Scale bar × 200 μm. (A) Control- normal structure of the kidney. (B) Triplavar treated- Presence of collagen fibers (C) *M. charantia* extract Low dose- histoarchitecture normal (D) *M*. *charantia* extract high dose- histoarchitecture essentially normal (E) *M. charantia* extract low dose and Triplavar- histoarchitecture essentially normal (F) *M. charantia* extract high dose and Triplavar- histoarchitecture essentially normal.

MT stains showed deposition of collagen fibers in the triplavar treated animals (Group B). These changes were absent in all groups treated with *M. charantia* and showed restoration of the histological layout similar to control (see Fig. 5).

## 4 Discussion

The potential impacts of HAART cannot be underscored despite challenging pitfalls with therapy in the reduction of mortality and morbidity. However, people living with HIV and AIDS (PLWHA) require consideration of organ-specific toxicities (especially kidney and liver) that can emanate from HAART use. Medicinal plants rich in phytochemical components has been shown to hold promise as an alternative therapy for the alleviation of some of the toxicities of HAART [9]. *M. charantia* possesses large amounts of phytochemical properties which are powerful counter measures against oxidative stress tissue damage [10]. The study, therefore, demonstrates the possible protective role of *M. charantia* in the mitigation of HAART’s ravages on the renal parameters.

From our study, derangements of the cytoarchitectural patterns of the kidney were seen as a result of treatment with HAART regimen (triplavar). These were characterized by tubular epithelial desquamation and glomerular capillary abnormalities (capillary wall occlusion and disruption of the capillary loops) with extracellular matrix accumulation which is suggestive that disturbances between the mesangial cell and glomerular capillaries may have been drastically altered. Glomerular abnormalities often result in a decrease in surface area available for filtration and a consequent decline in glomerular filtration rate and reduction in metabolic activity. Our observations in the H and E stains corresponded with PAS and MT staining intensities of the kidney sections and there is a supporting link. Photomicrographs of HAART- regimen (triplavar) showed vacuolation of tubules which are characterized by a high proportion of carbohydrates such as glycogen and glycoproteins and mild deposition of collagen fibers and hyaline substances.

These histological pathologies observed with HAART treated alone were ameliorated by *M. charantia* possibly due to its antioxidative properties as previously reported by [11,12]. Putatively, pathways for the generation of reactive oxygen species (ROS) leading to oxidative stress and toxicity of HAART relies on mitochondria-related perturbations that manifests in many side effects such as hepatic failure, testicular dysfunctions and renal disorders [10]. The ‘mitochondrial dysfunction hypothesis’ reviewed in [13] is believed to operate via energy deprivation, mitochondrial oxidative stress and consequent mitochondrial DNA damage. Co-administration of *M. charantia* and HAART mitigates the intensive histopathological changes of the rat kidney in our study by restoring the interstitial and restitution of the normal epithelial lining of the tubules. Our result corroborates with the findings of [14] on the histological assessment of the kidney following *M. charantia* leaf extract which shows normal histological layout when compared to normal rats.

The severity of oxidative damage depends on the extent of disturbances in the normal redox state. Excess ROS must be promptly eliminated from cells and this is done by a variety of antioxidant defense mechanisms. From our result, there were reductions in SOD and CAT levels in the groups treated with HAART alone when compared with control rats. This decline may precipitate into an oxidative stress status with consequent damage to cells and membranes in the renal tissue. However, the reversed levels on treatment with a low and high dose of *M. charantia*, and its co-administration with triplavar indicate an antioxidative therapeutic effect against HAART induced oxidative stress via significant enhancement of enzymatic antioxidant activities [15]. Our result also showed that reduced levels of GSH observed in triplavar treated group may be due to inactivation caused by reactive oxygen species. Administration of *M. charantia* extracts increased the levels of GSH in rats. HAART toxicity amplifies free radical formation through auto-oxidation of unsaturated lipids in plasma. The free radical produced may react with polyunsaturated fatty acids in cell membranes leading to lipid peroxidation [16]. Lipid peroxide-mediated tissue damage has been observed in PLWHAs under HAART. The increased lipid peroxidation in PLWHAs undergoing treatment with HAART may be due to observed remarkable increase in the concentration of TBARS and hydroperoxides in the kidney [17]. In our study, TBARS levels in the kidney were significantly high in triplavar treated group, but however, treatment with *M. charantia* extract and its co-administration with triplavar significantly lowered the level of TBARS compared to control.

Retention of renal electrolytes (sodium and potassium) is a manifestation of a functional overload of the nephron and is likely to contribute to elevated pressure in the vascular wall of the kidney which will eventually lead to organ hypertrophy and a decline in glomerular filtration causing leakage of albumin, podocytes effacement and loss of surface area available for filtration [18]. In our study, we observed leakage of albumin and renal electrolytes retention in the urine of triplavar treated rats. Indicating that glomerular endothelial barrier may have been altered. Although this observation was mild and manifested from the sixth week of our study and continued till the end of our study. Adjuvant treatment with *M. charantia* extract at both low and high dose was able to mitigate the abnormalities and restored the functions of the kidney to normal. Our result also shows urea retention in the rats that were treated with triplavar. Low concentration of urea in the kidney suggests a reduced turnover of protein and thus reflects in glomerular filtration rate and worsens renal function [19], more so, increased level of urea in the blood suggests enhanced amino acids fueled gluconeogenesis which leads to increased nitrogen load to the liver where urea is formed [20]. Administration of *M. charantia* fruit extract was able to restore the urinary metabolites to normal when compared to control rats.

As markers of renal function, BUN and serum SCr routinely serve as indicators for normal biological, pathologic processes, or pharmacologic responses to a therapeutic intervention. Our study revealed marked impairment in renal function with significantly raised levels of BUN and SCr concentrations in triplavar treated rats and this is consistent with lower BUN and SCr clearance. These elevations in levels of BUN and SCr concentrations might have resulted from remarkable leakage due to hypercellularity of the glomeruli and tubular degradation. Treatment with *M. charantia* fruit extract at both low and high dose prevented the development of nephrotoxicity by significantly lowering kidney injury markers such as BUN and SCr. The mitigation of kidney injuries due to increase in clearance of BUN and SCr by the kidney supports the report of [21] and further proves the biologically active components of the plant.

In our study we observed elevated levels of blood glucose in groups treated with HAART regimen (triplavar) indicating that HAART regimen suppresses glucose uptake by the tissues. This could occur as a result of complications arising from occlusion of the vascular wall and imbalance of endogenous vasodilators and constrictors [22]. HAART associated hyperglycemia is probably multifactor in its etiology, affecting glucose metabolism and insulin sensitivity, and altering the activity of glucocorticoid receptors in several tissues including the kidney. This promotes insulin resistance and impaired glucose tolerance through altered secretion of adipokines and other inflammatory markers such as interleukin 6, 8, 10 and macrophage chemotactic protein-1 [22]. Treatment with *M. charantia* extract significantly lowered blood glucose level. We speculate that the possible mechanism by which *M. charantia* decreases blood glucose level may be by potentiation of insulin effect by increasing either the pancreatic secretion of insulin from beta cells of islets of Langerhans. However, this claim is open for more studies to be conducted to provide any support.

Organ and body weight analysis offer insight on toxicity of test compounds in toxicology studies. In this study, there were significant decreases in body weight of rats treated with triplavar. These reductions may be attributed to morphological changes or protein wasting due to unavailability of carbohydrate for utilization of energy [23]. *M. charantia* extracts slowed the weight- gaining potential of the antiretroviral regimen as depicted in the results. The overall implication of this report agrees with the context of other results assessed in the present study. Although in our investigation, there was no significant difference in the weight coefficient (organ/body weight ratio) of kidney tissue in all groups. However, renal toxicity just like testicular toxicity can manifest in the form of organ hypertrophy.

## 5 Conclusion

Based on the results assessed in our study, *M. charantia* fruit extract prevented the pathological changes observed to a moderate extent. This might be due to pronounced antioxidant properties and anti-lipid peroxidation activities. In conclusion we therefore inferred that HAART induces toxicity of the kidney and *M. charantia* fruit extract has appreciable potentials to prevent damage to the kidney.

## Conflict of interest

The authors declared that there is no conflict of interest.
